# Photochemical advanced oxidative process treatment effect on the pesticide residues reduction and quality changes in dried red peppers

**DOI:** 10.1038/s41598-023-31650-4

**Published:** 2023-03-17

**Authors:** Ji-Yeon Bae, Deuk-Yeong Lee, Kyeong-Yeol Oh, Dong-Kyu Jeong, Dong-Yeol Lee, Jin-Hyo Kim

**Affiliations:** 1 Department of Agricultural Chemistry, Institutes of Agriculture and Life Science (IALS), Gyeongsang National University, Jinju, 52727 Republic of Korea; 2 Anti-Aging Research Group, Gyeongnam Oriental Anti-Aging Institute, Sancheong, 52215 Republic of Korea

**Keywords:** Environmental sciences, Risk factors

## Abstract

Pesticide residues in crops are widely monitored, and the residue reduction techniques at the post-harvest stage are important to maintain food safety. In dried crops, pesticide residues can be concentrated after dehydration, which increases concerns regarding residue risk. Therefore, the residue reduction effects of ultraviolet (UV), ozone, and photochemical advanced oxidative process (pAOP) were investigated for dried peppers at the post-harvest stage. UV_254_ treatment reduced 59.7% of the residue concentration on average, while UV_360_ showed a reduction of only 13.3% under 9.6 W m^−2^ of UV exposure for 24 h. Gaseous ozone treatments reduced the residue concentrations up to 57.9% on average. In contrast, the pAOP treatment reduced the concentration up to 97% and was superior to UV or ozone treatment alone. Increased drying temperature under pAOP condition resulted in higher reduction ratios at 40–80 °C. The pAOP conditions with 12 and 24 µmol/mol of ozone and UV_254_ irradiation for 24–48 h reduced the residue concentrations to 39–67%. Particularly, difenoconazole, fludioxonil, imidacloprid, and thiamethoxam residue concentrations were drastically reduced by over 50% under 12 µmol/mol ozone of the pAOP condition, while carbendazim, fluquinconazole, and pyrimethanil were relatively stable and their concentrations reduced below 50% under 24 µmol/mol ozone of the pAOP treatment. Various drying-related quality parameters of drying peppers such as water-soluble color, capsanthin, capsaicinoids, acid value, peroxide value, and thiobarbituric acid value were slightly altered, but not significantly, under 12 µmol/mol ozone of the pAOP condition, while the peroxide value was significantly altered under the higher ozone conditions. Therefore, pAOP treatment combined with gaseous ozone can be used for reducing residual pesticides in peppers without greatly reducing quality.

## Introduction

Pesticides are widely applied to the cultivation of food crops to increase crop productivity and quality. However, the pesticide usage in farms, preharvest residual limits, and maximum residue limits (MRLs) are strictly regulated by governmental organizations owing to their potential toxicity^1^. For most vegetables, the MRLs are established based on those in fresh crops, and these MRLs are applied to dried crops by considering the dehydration factors^1,2^.

Hot red peppers represent a source of vitamins, minerals, and phenolic antioxidants, and they are used as an ingredient in the form of dried powder for coloring and flavoring in spicy seasoning, sauce, and various foods^3,4^. Over 36 million tons of fresh peppers are produced worldwide annually, and they are dried via various techniques, including exposure to the sun, hot air, infrared and microwave radiation, and freeze drying, to extend their shelf-life while retaining high quality^4–7^. Among the drying methods, hot-air drying is most commonly used owing to its easy handling procedure, consistent efficiency, and for economic reasons. Thus, the optimization of hot-air drying process has been evaluated to increase the quality of dried crops, including colors, flavors, polyphenols contents, peroxide values (PVs), and acidity values (AVs), however, pesticide residue safety has not been investigated extensively^8–11^.

The residue levels of pesticides in crops may either decrease or increase after dehydration, depending on the thermal stability and vapor pressure of the applied pesticides. Volatile or unstable pesticides, including chlorpyriphos, dimethoate, fenitrothion, iprodione, malathion, methamidophos, and methidathion, tended to exhibit decreased residue levels after drying^8,12,13^. In contrast, non-volatile and thermostable pesticides, such as benalaxyl, biteranol, metalaxyl, phosalone, and vinclozolin, showed an increase in residue levels in dried crops due to water loss in the fruits during the dehydration process^8,14–16^. The increase in residue levels in dried crops increases the dietary exposure risk, consequently lowering food safety^14,17^. Thus, the residue reduction of thermostable pesticides in the postharvest process of dried crops is urgently required. Recently, a few residue level-reduction treatments were studied for use as post-harvest treatments, including storage and washing; however, they cannot effectively reduce the levels of systemic and biochemically stable pesticides^3^. Oxidative treatments using UV radiation or ozone treatment are generally considered as alternative treatments for use in the post-harvest process^17–21^. The absorption of UV and visible light of pesticide molecules activates the nonbonding and bonding electrons in the molecule and spontaneously initiates the oxidative reaction with oxygen and water in the presence of atmospheric air. In particular, vacuum ultraviolet (VUV, < 200 nm) treatment can activate the electrons of covalent bonds in oxygen molecules and generate ozone, HO·, hydrogen peroxide, singlet oxygen, and other reactive oxygen species in air^21–25^. These oxidative treatments that occur in air can stimulate the oxidative degradation and decomposition of organic compounds, including pesticides and nutrients^26–28^.

Recently, the effects of UV irradiation on pesticide residue reduction were studied on several commercial pesticides, including acetamiprid, carbofuran, diuron, isoproturon, alachlor, atrazine, and chlorfenvinphos, among others^29–31^. And ozone treatments were studied for difenoconazole, chlorpyrifos, pyraclostrobin, pyrimethanil, azoxystrobin, and linuron, among others^32,33^. UV treatment is effective only in the degradation of pesticide residues that are present on the surface of crops, while the degradation capability of ozone is restricted to certain types of pesticides^34,35^. Furthermore, ozone is retained in the processed crops after the aqueous ozone treatment. Thus, some stable pesticides resistant to oxidation and UV radiation, such as triazole class pesticides, require higher degradation power for removing the residues^22,36^.

**Figure 1:**
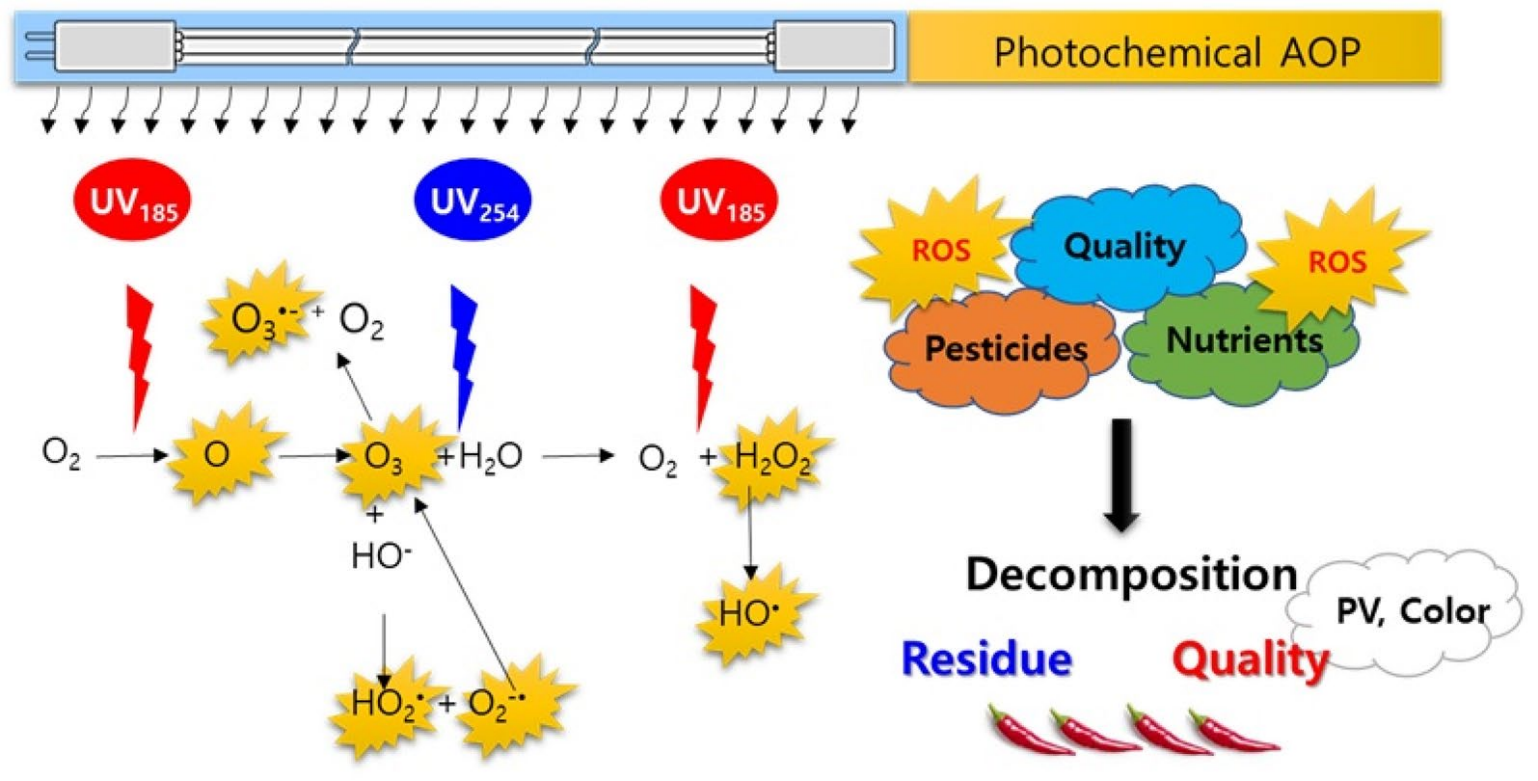
ROS generation and the oxidative degradation of food chemicals under pAOP process.

An advanced oxidation process was recently applied to wastewater treatment to remove organic contaminants and was known as a more potent oxidative treatment than ozone or UV. This is a combined treatment with an oxidant (ozone, oxygen, and hydrogen peroxide) and a radical initiator (UV, ultrasound, Fe^2+^, TiO_2_, and alkaline) in aqueous conditions^37–40^. Thus, this process can be considered the post-harvest treatment for crops, at the washing step; however, this process is not commonly used because it would create ozone residue, decrease the crop quality, and increase the exposure risk of reactive oxygen species (ROS) to workers^39,41^. However, the photochemical advanced oxidation process (pAOP) without metal catalysts, combined with UV and gaseous ozone may be feasible for use in the drying step in a closed chamber, without concerns of ozone residue. Herein, pAOP treatment effects on pesticide residue-level reduction and on crop quality parameters were investigated in peppers, and the acceptable pAOP conditions and most sensitive quality parameters for dried red peppers were suggested (Fig. 1). All the tested pesticides were frequently detected in dried peppers based on the low vapor pressure with thermostable compounds among the allowed pesticides.

## Results and discussion

### UV irradiation effect on the pesticide residue-level reduction on paper disc

To confirm the residue stability of the tested pesticides (carbendazim, dimethomorph, fluquinconazole, thiamethoxam, imidacloprid, tetraconazole, and myclobutanil) under hot-air drying condition, the reduction ratio was measured at a temperature generally accepted for drying crops (40 °C and 60 °C) and at 80 °C. The reduction ratios of all the tested pesticides remained over 73% at 40–80 °C after thermal treatment for 24 h (Table S1). The thermal stability was sufficient for facilitating the investigation of UV, ozone, and pAOP treatment effects on the pesticide residue at the conventionally accepted temperature in hot-air drying of peppers.

To confirm the UV stability of the pesticides, all pesticides were exposed to UV radiation at 360 nm or 254 nm for 24 h under a UV lamp (9.6 W/m^2^) at 60 °C. All pesticides were stable under UV-A radiation at 360 nm except for imidacloprid (31% reduction) and thiamethoxam (28% reduction), while all pesticides under UV_254_ irradiation were decomposed and showed reduction ratios of 49–89% except for carbendazim which had a reduction ratio ≤ 20% (Table 1). UV_254_, possessing greater energy than UV_360_, can more easily excite π-bonded electrons in the pesticide molecule, thereby stimulating the oxidative decomposition of pesticides by oxygen and water in the surrounding air^42^. As a result, a high pesticide residue reduction ratio was shown in UV_254_. Thus, UV_254_ irradiation represents an effective process for pesticide residue-level reduction; however, systemic pesticides and pesticides which were deposited on shading surfaces not exposed to radiation may not be degraded using this process^34,35^. Thus, oxidants treatment using ozone and hydrogen peroxide can be considered for an effective residue-level reduction in crops.

**Table 1:** The selected pesticide residue reduction ratio after the treatment of UV for 24 h at 60 °C^a)^.

	Residual pesticide reduction ratio (%)	
	UV_360_ (9.6 W/m^2^)	UV_254_ (9.6 W/m^2^)
Carbendazim	< 10^a^	20 ± 0.8^b^
Dimethomorph	16 ± 0.8^b^	49 ± 2.0^c^
Fluquinconazole	< 10^a^	52 ± 2.9^b^
Imidacloprid	31 ± 1.3^b^	86 ± 0.8^c^
Myclobutanil	18 ± 1.1^a^	63 ± 1.1^b^
Tetraconazole	< 10^b^	58 ± 1.3^c^
Thiamethoxam	28 ± 1.4^b^	89 ± 0.8^c^
Average	13.3^a^	59.7^b^

Different superscript letters represent the result of tukey test (*p* < 0.05).

### Residual pesticide reduction effect on gaseous ozone treatment

The oxidative degradation of pesticides through ozone treatment was evaluated at ozone concentrations ranging from 12 to 85 µmol/mol within the drying chamber. The gas effectively decomposed and reduced the concentrations of dimethomorph, imidacloprid, and thiamethoxam to reduction ratios of 67–93%, 24–85%, and 26–87% under 12–85 µmol/mol of ozone for 24 h at 60 °C, respectively, whereas carbendazim, fluquinconazole, tetraconazole, and myclobutanil stably remained at > 87%, > 61%, > 52% and > 59% after the ozone treatment, respectively (Table 2). Higher ozone concentrations were found to result in increased pesticide reduction ratios. However, the efficacy of ozone in reducing the levels of triazole-class pesticides was limited at ozone concentrations below 85 µmol/mol. Thus, more potent oxidative conditions, such as in pAOP with UVC and ozone, than those in ozone treatment were considered to reduce the pesticide residue levels in crops.

**Table 2:** The selected pesticide residue reduction ratio after the treatment of ozone for 24 h^a)^.

	Residue reduction ratio (%)			
	Ozone concentration			
	12 µmol/mol	24 µmol/mol	45 µmol/mol	85 µmol/mol
Carbendazim	< 10^a^	< 10^a^	< 10^a^	13 ± 2.8^b^
Dimethomorph	67 ± 2.2^a^	84 ± 4.2^b^	93 ± 1.8^c^	92 ± 1.2^c^
Fluquinconazole	< 10^a^	< 10^a^	22 ± 2.0^b^	39 ± 2.5^c^
Imidacloprid	24 ± 3.5^a^	38 ± 2.9^b^	50 ± 2.7^c^	85 ± 1.7^d^
Myclobutanil	< 10^a^	< 10^a^	20 ± 2.1^b^	41 ± 1.1^c^
Tetraconazole	< 10^a^	< 10^a^	< 10^a^	48 ± 2.3^b^
Thiamethoxam	26 ± 1.8^a^	42 ± 3.7^b^	58 ± 1.7^c^	87 ± 1.2^d^
Average	16.7^a^	23.4^a^	34.7^a^	57.9^b^

Different superscript letters represent the result of tukey test (*p* < 0.05).

### Residual pesticide reduction on the paper disc by pAOP treatment with UV_254_ and ozone

**Figure 2:**
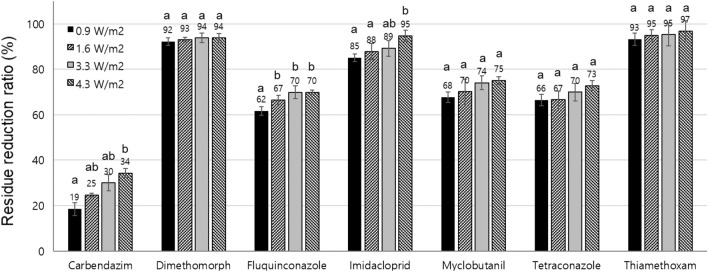
Comparison of pesticide residue reduction on the paper disc by the UV irradiation under the pAOP condition with 45 µmol/mol of ozone and UV_254_ at 60 °C for 24 h (tukey test, *p* < 0.05).

pAOP treatment comprising UV_254_ irradiation and ozone can stimulate oxidation and degrade the pesticides. Using four different UV_254_ irradiation energies (0.9, 1.6, 3.3 and 4.3 W m^−2^), the pesticide reduction ratios were investigated under 45 and 85 µmol/mol of gaseous ozone conditions. All the tested pesticides treated with pAOP showed higher reduction ratios than that of ozone or UV_254_ irradiation alone (Tables 1 and 2), and a higher UV_254_ irradiation facilitated a higher residue reduction ratio (Fig. 2). Dimethomorph, imidacloprid, and thiamethoxam that were sensitive to UV or ozone treatment decomposed readily and their concentrations reduced to reduction ratios over 85% in the AOP conditions under 45 µmol/mol; the reduction ratios were almost two-fold higher than that of the individual treatment with UV_254_ or ozone. Furthermore, fluquinconazole, myclobutanil, and tetraconazole that were relatively stable under UV_254_ irradiation or ozone treatment showed residue reduction ratios of 62–70%, 68–75%, and 66–73% under 45 µmol/mol ozone of the pAOP condition, respectively.

Carbendazim which was the most stable pesticide under ozone or UV_254_ irradiation showed a reduction ratio of up to 34%. When the ozone concentration was increased to 85 µmol/mol under 4.3 W/m^2^ of UV_254_ irradiation, carbendazim concentration was reduced to a ratio of 52%, and the other tested pesticide was reduced to a ratio of over 90% (Table S2).

**Figure 3:**
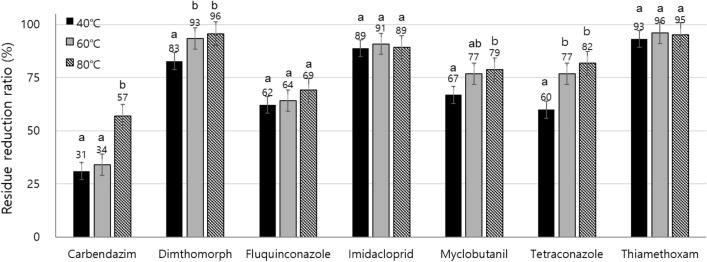
Residual pesticide reduction ratio on the paper disc at three different temperatures (40, 60 and 80 °C) by the pAOP condition with 45 µmol/mol ozone and 4.3 W m^-2^ UV_254_ irradiation (tukey test, *p* < 0.05).

The residue reduction ratios were evaluated at different temperatures, including 40, 60 and 80 °C, under 45 µmol/mol ozone and 4.3 W m^−2^ UV_254_ irradiation, and a relatively higher temperature could stimulate the oxidative decomposition of pesticides under the AOP condition (Fig. 3). The thermal effect on residue reduction for carbendazim drastically increased by the reduction ratios to 57% at 80 °C, and the reduction ratios for the pesticides appeared to increase at higher temperatures.

### Pesticide residue reduction in red peppers under pAOP conditions

For the application of pAOP condition to red peppers, the drying temperature was controlled to below 80 °C which is conventionally accepted for drying peppers owing to the prevention of changes in quality^3,4^. The two pAOP conditions that were 45 and 85 µmol/mol ozones with UV_254_ irradiation (4.3 W m^−2^) were initially applied for drying red peppers at 60 °C for 24 h and 48 h. However, the color of the dried red pepper changed substantially to brown in the sensory analysis; thus a weaker pAOP condition than the initial candidates was required to minimize the quality changes in the red pepper. The ozone concentration was decreased to 12, 18 or 24 µmol/mol, while UV_254_ irradiation power was increased to 9.6 W m^−2^ for pAOP treatment. Under these adjusted AOP conditions, the sensory color change in dried red peppers did not show in comparison with the control after 48 h of treatment. Thus, the residue reduction and quality changes of the dried pepper were tested under the adjusted pAOP conditions (Fig. S1).

The highest pesticide reduction was found after treatment with 24 µmol/mol ozone with 9.6 W m^−2^ of UV_254_ irradiation for 48 h as expected (average 67%); thiamethoxam, imidacloprid, fludioxonil, and difenoconazole concentrations were reduced to over 81% (Table 3). The residue reduction ratios for other pesticides were decreased in the order of dimethomorph (72%), tebuconazole (68%), tetraconazole (66%), myclobutanil (60%), fluquinconazole (45%), pyrimethanil (43%), and carbendazim (40%). The lowest residue reduction ratio was found in the pAOP condition of 12 µmol/mol of ozone and UV_254_ irradiation for 24 h (average 39%). Carbendazim, dimethomorph, myclobutanil, fluquinconazole, tebuconazole, and pyrimethanil were reduced below 38% under the condition. Under the other pAOP conditions such as 12 µmol/mol of ozone for 36–48 h, 18 µmol/mol of ozone for 24–36 h, and 24 µmol/mol of ozone for 24 h, the average reduction ratios of all tested pesticides were 44–52% similarly. Fludioxonil as a phenylpyrrole group pesticide showed the highest residue reduction ratio (over 85%), and neonicotinoidal thiamethoxam, and imidacloprid showed ≥ 40% of reduction ratio under all pAOP conditions, whereas carbendazim, fluquinconazole, and pyrimethanil were stable under the adjusted AOP conditions (reduction ratio ≤ 45%) (Table 3).

**Table 3:** Residual pesticide reduction in dried red pepper after the pAOP treatments^a)^.

Pesticide	Residue reduction (%)								
	12 µmol/mol ozone			18 µmol/mol ozone			24 µmol/mol ozone		
	UV 24 h	UV 36 h	UV 48 h	UV 24 h	UV 36 h	UV 48 h	UV 24 h	UV 36 h	UV 48 h
Carbendazim	< 10^a^	15 ± 7.7^ab^	25 ± 11^ab^	15 ± 15^ab^	22 ± 8.9^ab^	30 ± 6.7^b^	30 ± 6.8^bc^	35 ± 4.4^bc^	40 ± 7.8^c^
Difenoconazole	50 ± 14^a^	55 ± 7.3^a^	65 ± 7.7^ab^	58 ± 6.9^ab^	62 ± 6.2^ab^	68 ± 7.4^bc^	69 ± 8.7^bc^	75 ± 5.3^bc^	81 ± 8.6^c^
Dimethomorph	23 ± 15^a^	35 ± 14^ab^	45 ± 13^ab^	40 ± 13^ab^	50 ± 9.9^b^	68 ± 9.6^bc^	54 ± 8.3^b^	65 ± 8.4^bc^	72 ± 8.3^c^
Fluquinconazole	21 ± 14^a^	27 ± 11^a^	30 ± 7.1^ab^	22 ± 14^a^	31 ± 8.0^ab^	35 ± 7.8^ab^	24 ± 10^ab^	38 ± 7.9^ab^	45 ± 7.9^b^
Fludioxonil	85 ± 10^a^	87 ± 8.0^a^	90 ± 7.8^a^	88 ± 5.1^a^	90 ± 5.6^a^	92 ± 6.5^a^	89 ± 5.6^a^	92 ± 3.3^a^	95 ± 4.2^a^
Imidacloprid	65 ± 8.4^a^	68 ± 8.1^a^	70 ± 6.4^ab^	68 ± 7.3^a^	72 ± 8.1^ab^	75 ± 10^ab^	73 ± 7.5^ab^	78 ± 9.7^ab^	83 ± 6.0^b^
Myclobutanil	33 ± 12^a^	36 ± 14^a^	40 ± 15^a^	35 ± 11^a^	43 ± 9.5^ab^	50 ± 9.9^ab^	38 ± 12^a^	45 ± 8.9^ab^	60 ± 10^b^
Pyrimethanil	27 ± 10^a^	32 ± 5.4^ab^	38 ± 7.9^ab^	30 ± 4.2^ab^	34 ± 10^ab^	40 ± 8.9^ab^	33 ± 11^ab^	38 ± 6.1^ab^	43 ± 6.3^b^
Tebuconazole	38 ± 14^a^	40 ± 7.5^a^	45 ± 13^a^	40 ± 9.9^a^	42 ± 9.5^a^	48 ± 8.3^a^	48 ± 14^a^	58 ± 7.8^ab^	68 ± 5.9^b^
Tetraconazole	45 ± 10^a^	49 ± 6.2^ab^	55 ± 5.2^ab^	45 ± 4.0^a^	52 ± 8.6^ab^	58 ± 12^ab^	45 ± 15^a^	55 ± 6.2^ab^	66 ± 6.3^b^
Thiamethoxam	40 ± 6.2^a^	48 ± 7.3^ab^	60 ± 6.8^b^	45 ± 8.9^ab^	55 ± 9.1^b^	65 ± 8.4^bc^	69 ± 9.3^bc^	75 ± 6.7^c^	82 ± 4.3^c^
Average	39^a^	47^ab^	51^ab^	44^ab^	50^ab^	57^bc^	52^b^	59^bc^	67^c^

Different superscript letters represent the result of tukey test (*p* < 0.05).

**Figure 4:**
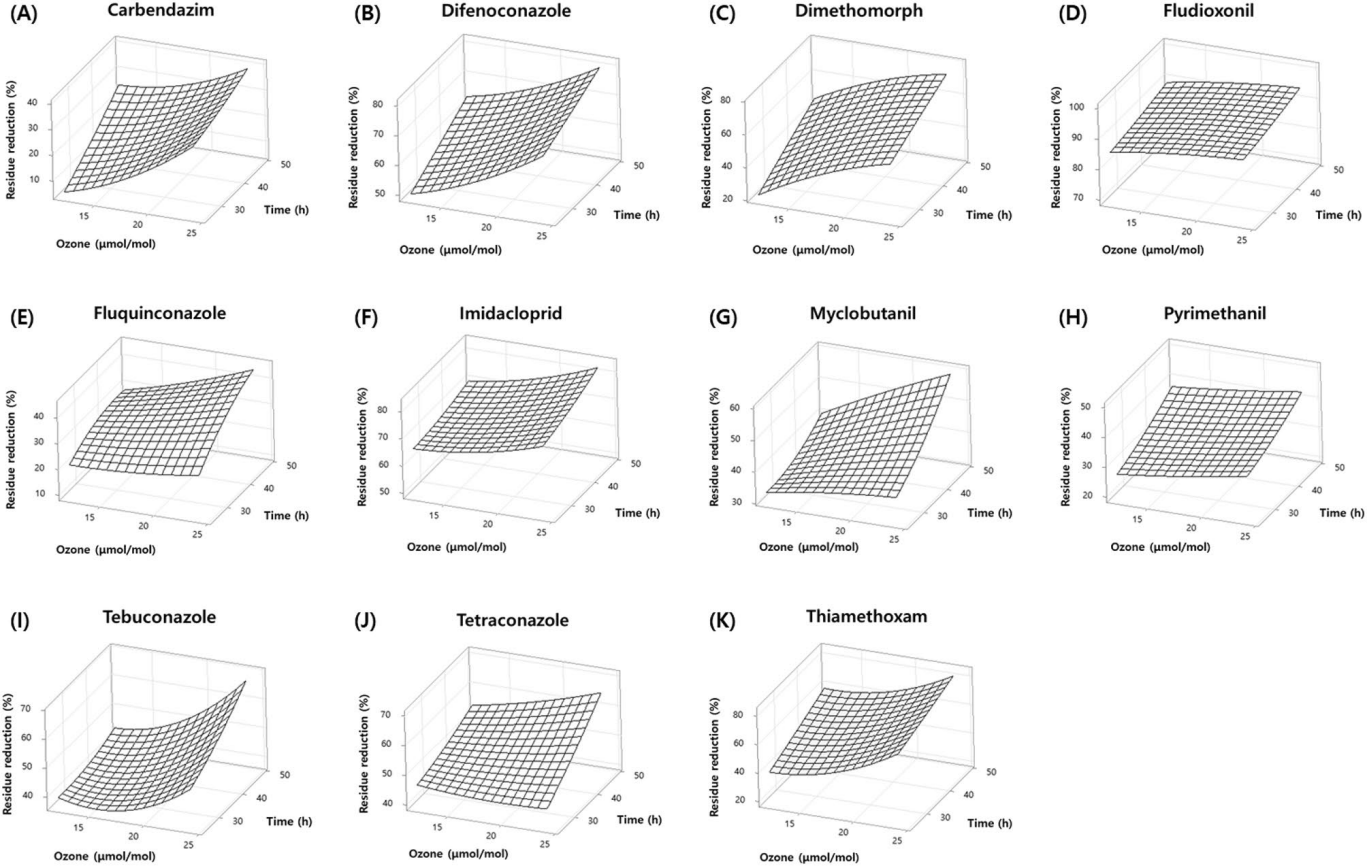
Three-dimensional response surface plots for the reduction ratios of carbendazim (**A**), difenoconazole (**B**), dimethomorph (**C**), fludioxonil (**D**), fluquinconazole (**E**), imidacloprid (**F**), myclobutanil (**G**), pyrimethanil (**H**), tebuconazole (**I**), tetraconazole (**J**), and thiamethoxam (**K**) by ozone concentration and the treatment time under pAOP condition.

The response surface plots showed a pronounced increase as the concentration of ozone and treatment time increased for the residue reduction ratio (Fig. 4). Ozone concentration and time for the pAOP treatment showed a significant influence on the experimental response, and the interaction of ozone concentration and treatment time interaction was statistically significant except for fludioxonil (Table S3). Among the tested pesticides, carbendazim, difenoconazole, dimethomorph, and thiamethoxam were found to be more sensitive to variations in ozone concentration during pAOP treatment, whereas fluquinconazole, myclobutanil, tebuconazole, and tetraconazole exhibited greater sensitivity to treatment duration, resulting in substantial reductions in residual levels.

### Quality changes in the dried red pepper after pAOP treatment

**Figure 5:**
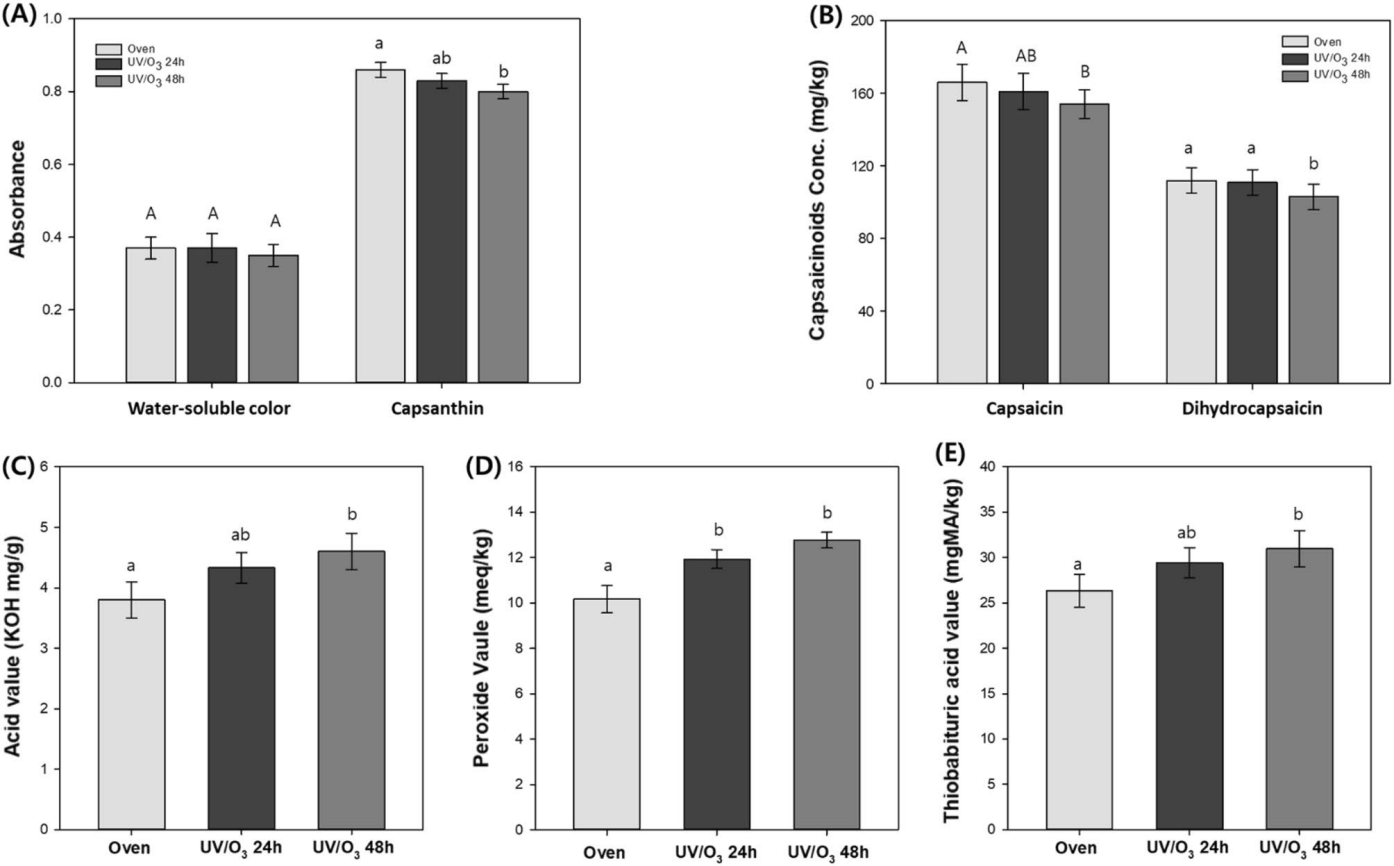
Quality changes of the dried red peppers for (**A**) water-soluble color and capsanthin, (**B**) capsaicin and dihydrocapsaicin, (**C**) AV, (**D**) PV and (**E**) TBAV by the AOP treatments with 24 µmol/mol of ozone with 9.6 W m^−2^ of UV_254_ irradiation (tukey test, *p* < 0.05).

Changes in the quality of the dried red peppers were compared based on to water-soluble color, capsanthin, capsaicinoids, PV, AV, and thiobarbituric acid value (TBAV). The water-soluble color of the dried red pepper showed no major change after all the pAOP treatments. However, the capsanthin and capsaicinoids levels significantly decreased by 7, and 8%, and AV, PV, and TBAV increased by 21, 26 and 17% after pAOP treatment under 24 µmol/mol ozone for 48 h, respectively (*p* < 0.05) (Fig. 5). The quality parameters of the peppers treated under the AOP conditions with 24 µmol/mol ozone for 24 h, with 18 µmol/mol ozone for 24 h, and with 12 µmol/mol ozone for 48 h were slightly altered, but not significantly, except for PV. In addition, all quality parameters were not significantly altered after pAOP treatment under 12 µmol/mol of ozone for 36 h (*p* > 0.05).

Ozone concentration in the pAOP condition was revealed as a key factor for pesticide residue reduction and quality change of crops. Although the stronger pAOP conditions with relatively longer treatment time showed higher pesticide reduction ratios in the red peppers, considering the quality changes of red peppers, the pAOP treatment conditions using 12 µmol/mol of ozone with UV_254_ irradiation up to 48 h were recommended. And carbendazim was the most stable residue, whereas the neonicotinoid insecticides readily reduced the residue in the pepper. In addition, PV among the quality parameters for crops was the most sensitive under oxidative drying conditions, and it should be considered to optimize pAOP treatment conditions in crop drying.

## Materials and methods

### Chemicals

Analytical standards of thiamethoxam, imidacloprid, carbendazim, dimethomorph, fludioxonil, myclobutanil, fluquinconazole, tetraconazole, tebuconazole, difenoconazole, and pyrimethanil were used Dr. Ehrenstorfer™ from LGC Limited (Manchester, NH, USA). The physico-chemical properties of the tested pesticides were described in Table S4. Capsaicin and dihydrocapsaicin were purchased an analytical grade from Sigma-Aldrich® of Merck KGaA (Darmstadt, Germany). Acetone, acetonitrile, benzene, chloroform, methanol, ethanol, and water were purchased HPLC grade from Merck KGaA (Darmstadt, Germany). Reagent grade acetic acid, formic acid, ammonium formate, diethyl ether, sodium chloride, magnesium sulfate anhydrous, sodium acetate, potassium hydroxide, sodium thiosulfate, and thiobarbituric acid were purchased from Sigma-Aldrich®.

### Ozone and UV_254_ treatment chamber

**Figure 6:**
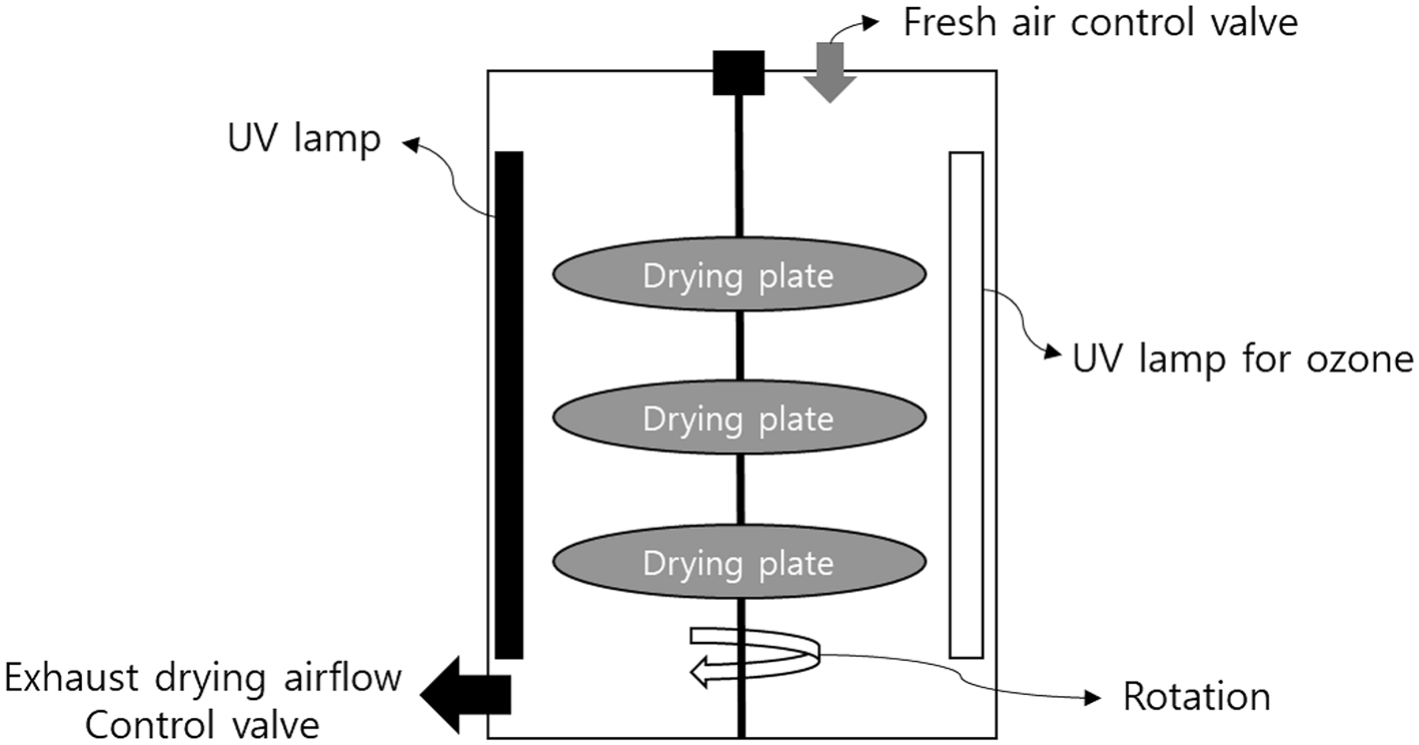
Ozone and UV treatment chamber for crop drying.

Ozone and UV_254_ irradiation experiments were performed in a mechanical convection-type drying oven that contained UV lamps. The temperature was controlled to 40, 60 and 80 °C in the chamber, and UV light sources were controlled at 254 nm and 360 nm; ozone was generated by the VUV lamp installed in the oven. All the UV lamps were purchased from Hansung Ultraviolet Co. Ltd. (Seongnam, Republic of Korea). The pAOP treatments for pepper were performed in a re-designed convection-type oven (1.15 × 0.6 × 0.65 m, 0.448 m^3^; Fig. 6). For a similar UV exposure condition, the drying plate was rotated at 5–10 rpm. After the ozone and UV_254_ treatment, the drying chamber was operated until the peppers were dried entirely for up to 2 d, and the total operation time was 3 d.

### Pesticide degradation on paper disc in the oven

The pesticide degradation experiments were performed for seven selected pesticides (carbendazim, dimethomorph, fluquinconazole, imidacloprid, myclobutanil, tetraconazole, and thiamethoxam) that are frequently detected in pepper. Briefly, 20 µL of pesticide standard solution (100 µg mL^-1^) was loaded on a paper disc (8 mm, Advantec®, Toyo Roshi Kaisha, Ltd., Japan). The disc was placed under a UV lamp in the oven for the UV exposure experiment. The distance from the light source was set based on the selected power (0.9–9.6 W/m^2^), and the UV_254_ irradiation power was measured with Portable Luxmeter (HD2102.1, Delta OHM S.r.l., Selvazzano Dentro, Italy). For the ozone exposure experiment, the disc was placed on the dark side in the oven, then the disc was dried under the generated ozone gas in the oven. The tested ozone (12–85 µmol/mol of ozone) was saturated within 10 min in the chamber. Ozone concentration in the oven was monitored using Ozone Monitor™ (Model 202, 2B Technologies, Boulder, CO, USA). The tested temperature was 40–80 °C. The dried disc was extracted with 1.0 mL acetonitrile in a microtube and filtered through a 0.23 µm of PTFE syringe filter and then instrumentally analyzed. All experiments were performed with five replications.

### Preparation of the pesticide-contaminated pepper

The tested pesticides were selected among the registered pesticides for pepper by the Korean government, including carbendazim, difenoconazole, dimethomorph, fludioxonil, fluquinconazole, imidacloprid, myclobutanil, pyrimethanil, tebuconazole, tetraconazole, and thiamethoxam (Table S5). The commercial pesticides were diluted with water following the good agricultural practice manual by Korea Crop Protection Association^43^, and 2 kg of fresh red peppers were dipped in the solution and then stored for 24 h in a fume hood at room temperature. The fresh peppers were dried in a drying chamber for three days to fully dry. During the total three days of drying, each of the treatments was performed for 24–48 h. All the drying experiments were performed in triplicate. The dried peppers were ground with dry ice and stored at − 20 °C until analysis.

### Sample preparation for the residue analysis in dried red pepper

The residue analysis method was followed by Song et al.^44^. Briefly, the ground pepper (2.0 g) was added to 10 mL of distilled water (DW) and then shaken for an hour at room temperature. Subsequently, 6 g MgSO_4_, 1.5 g NaOAc, 1.5 g NaCl, and 20.0 mL acetonitrile were added following vigorously shaking for an hour. The extract was sonicated for 10 min and centrifuged at 3000 g at 4 °C for 10 min. Subsequently, 1.0 mL of the supernatant was added to 150 mg MgSO_4_, and 50 mg primary-secondary amine resin (PSA). The mixture was vortexed and centrifuged at 8000 g at 4 °C for 10 min, and then filtered with 0.23 µm of PTFE syringe filter. The filtrate was analyzed with liquid chromatography-triple quadruple mass spectroscopy (LC–MS/MS, Agilent Co. Ltd., Santa Clara, CA, USA) for the quantitative analysis of carbendazim, difenoconazole, dimethomorph, fluquinconazole, imidacloprid, pyrimethanil, and thiamethoxam. For the analysis of fludioxonil, myclobutanil, tebuconazole, and tetraconazole, 1.0 mL supernatant of the extracts was added to 150 mg MgSO_4_, 50 mg PSA, and 50 mg C18 following vortexing and centrifugation at 8000 g at 4 °C for 10 min. The supernatant was filtered with a syringe filter, and then analyzed with gas chromatography-mass spectroscopy (GC–MS, QP2020, Shimadzu Co., Japan).

### Instrumental analysis with GC–MS

For the quantitative analysis of fludioxonil, myclobutanil, tebuconazole, and tetraconazole, the separation was performed with Rtx™-5MS capillary column (Restek Co., Bellefonte, PA, USA) and the target ions were detected in selected ion mode. The quantitative and qualitative ions are presented in Table S6.

### Instrumental analysis with LC–MS/MS

For the quantitative analysis of carbendazim, difenoconazole, dimethomorph, fluquinconazole, imidacloprid, pyrimethanil, and thiamethoxam, the separation was performed with Poroshell 120 EC-C18 column (2.1 × 100 mm, 2.7 µm, Agilent Technologies Inc., Santa Clara, CA, USA). The mobile phase included DW and acetonitrile containing 0.1% formic acid and 5 mM ammonium formate. The ionization mode was electrospray ionization-positive and the target ions were detected in multiple reaction monitoring (MRM) mode. The quantitative and qualitative ions are presented in Table S7.

### Method validation for quantitative analysis of the pesticide

The residue analysis method was validated by determining the recoveries associated with relative standard deviation (RSD) of each pesticide at concentrations of 0.02 and 0.20 mg kg^−1^ in dried red pepper. The recoveries of the tested pesticides were 81.1–101.3% and the interday precisions ranged from 6.7 to 9.4%. The linearity of the standard curve for each pesticide was acceptable for quantitation (R^2^ > 0.999); the concentration ranges were 0.01–1.0 µg mL^−1^ for GC–MS analytes and 0.001–0.10 µg mL^−1^ for LC–MS/MS analytes. The method limit of quantitations for all tested pesticides were 0.01 µg kg^−1^ for dried red pepper.

### Pesticide residue reduction ratio

The residue-level reduction ratio was calculated from the comparison of UV irradiation and ozone treatment with no treatment.

$$ Reduction \, ratio \left( \% \right) = \left( {1 - \frac{{\left( {Concentration\,after\,treatment} \right)}}{{\left( {Concentration\,after\,no\,treatment} \right)}}} \right) \times 100 $$

### Data processing

The data statistical analysis was performed using the statistical program R (ver. 4.2.3, The R Foundation) was used. The optimization experiment was carried out using response surface methodology (RSM) for ozone concentration and time of pAOP treatment with Minitab (Version 18, Minitab Inc., State College, PA, USA).

### Color changes in dried red pepper

Water-soluble color changes in the pepper were evaluated by measuring non-enzymatic browning of the dried pepper. Non-enzymatic browning value was measured by the method of Rhim and Hong ^45^. Briefly, the powdered pepper (1.0 g) was suspended in 50 mL of DW, and water-soluble pigments were extracted at 30 °C for 4 h, centrifuged at 3000 g*,* and then the supernatant was filtered with a syringe filter (0.45 µm). The absorbance of the filtrate at 420 nm was measured using a visible spectrophotometer (Genesys 20, Thermo Fisher Scientific Inc., Waltham, MA, USA). For measuring the changes in capsanthin contents, the analysis method from the American Spice Trade Association (ASTA) color index for the dried pepper was followed. Briefly, the dried sample (1.0 g) was extracted with 40 mL acetone in an orbital shaker, and then centrifuged and filtered. The filtrate was diluted to tenfold with acetone and then the absorbance at 460 nm was measured using a spectrophotometer.

### Capsaicinoid contents in dried red pepper

The analysis method for capsaicinoids contents was slightly modified from the previous report by Othman et al.^46^. Briefly, capsaicin and dihydrocapsaicin were extracted with 95% ethanol (10 mL) from 1.0 g of the dried red pepper. Extraction was performed for 30 min on an orbital shaker at room temperature, and then the flask was warmed to 60 °C for 2 h and stirred for 30 min. The extract was centrifuged at 3000 g and then 1 mL of the supernatant was filtered with a syringe filter. The filtrate was analyzed using HPLC-UVD. The analytical instrument conditions were described in Table S8 in the supplemental information. The linearities of capsaicin and dihydrocapsaicin were > 0.999 for 2.0–200 mg L^−1^, and the RSDs for repetition were < 10%.

### Acid value (AV) in dried red pepper

Twenty gram of dried pepper sample was extracted with 400 mL diethyl ether for 4 h at room temperature and the extract was evaporated. The residue oil was refrigerated in a dark tight-stoppered glass bottle until analysis of AV and lipid oxidation. The AV of the pepper oil was determined using a volumetric titration method and the value was expressed as the amount of potassium hydroxide (mg) required to neutralize the free fatty acids present in 1 g of fat^47,48^. Briefly, 100 mL of the mixture of ethanol/diethyl ether (1/2, *v/v*) was added to 1.0 g of the oil sample following addition of a few drops of phenolphthalein indicator, and then the solution was titrated with 0.1 N KOH in ethanol.

$$ {\text{AV }}\left( {{\text{KOH mg}}/{\text{g}}} \right) = { }\frac{{\left( {V_{treatment} - V_{control} } \right) \times 5.611 \times F}}{Sample \left( g \right)} $$

V_treatment_ and V_control_ was the consumption volume of 0.1 N KOH in the treatment and control samples, and F was a factor of 0.10 N KOH.

### Lipid oxidation

PV was determined using the iodometric method according to the standard method for oil analysis of MFDS^48^. Briefly, the oil sample (1.0 g) was dissolved with chloroform/acetic acid (3/2, *v/v*) mixture in the presence of saturated KI, then titrated with 0.01 N Na_2_S_2_O_3_ using starch as a colorimetric indicator. And the results were expressed in milliequivalent peroxide/kg oil (meq/kg oil).

$$ {\text{PV }}\left( {{\text{meq}}/{\text{kg}}} \right) = \frac{{\left( {V_{treatment} - V_{control} } \right) \times F \times 0.01}}{Sample \left( g \right)} \times 1000 $$

V_treatment_ and V_control_ were the consumption volume of 0.01 N Na_2_S_2_O_3_ in the treatment and control samples, F was a factor of 0.010 N Na_2_S_2_O_3_.

The TBAV was measured with the method described in previous reports^49,50^. 1.0 g sample in benzene (10 mL) was added 1% thiobarbituric acid (10 mL) in 20% trichloroacetic acid then reacted at 95 °C for 30 min. The reaction mixture was measured absorbance at 530 nm and expressed as mg malonaldehyde (MDA)/kg.

$$ {\text{TBAV }}\left( {{\text{mg MDA}}/{\text{kg}}} \right) = \frac{{\left( {Abs_{treatment} - Abs_{control} } \right) \times D \times 100}}{Sample \left( g \right)} $$

Abs_treatment_ and Abs_control_ were the absorbances at 530 nm of the treated and control samples respectively, and D was the dilution factor of a sample.

## Supplementary Information

Supplementary Information.

## Data Availability

All data generated or analyzed during this study are included in this published article.
