# Supplementary material for: Photochemical advanced oxidative process treatment effect on the pesticide residues reduction and quality changes in dried red peppers
Source: Sci Rep. 2023 Mar 17;13:4444. doi: 10.1038/s41598-023-31650-4 (PMC10023666; doi:10.1038/s41598-023-31650-4)
Supplement: Supplementary file 1 — Supplementary Information. [file 41598_2023_31650_MOESM1_ESM.docx]

**Supporting Information**

**Photochemical advanced oxidative process treatment effect on the pesticide residues reduction and quality changes in dried red peppers**

Ji-Yeon Bae^1^, Deuk-Yeong Lee^1^, Kyeong-Yeol Oh^1^, Dong-Kyu Jeong^1,2^, Dong-Yeol Lee^1,2^, Jin-Hyo Kim^1,*^

*^1^ Department of Agricultural Chemistry, Institutes of Agriculture and Life Science (IALS), Gyeongsang National University, Jinju, 52727, Republic of Korea*

*^2^ Anti-Aging Research Group, Gyeongman Oriental Anti-Aging institute, Sancheong 52215, Republic of Korea*

**Table S1.** The selected pesticide residue reduction ratio after the thermal treatment for 24h.

|  | Residual pesticide reduction ratio (%) | | |
| --- | --- | --- | --- |
|  | 40℃ | 60℃ | 80℃ |
| Carbendazim  Dimethomorph  Fluquinconazole  Imidacloprid  Tetraconazole  Thiamethoxam  Myclobutanil | <10%  <10%  <10%  <10%  <10%  <10%  <10% | <10%  <10%  <10%  12±4.6%  13±5.3%  15±4.8%  16±3.6 | <10%  12±4.2%  13±5.3%  27±4.3%  15±5.7%  26±7.2%  18±4.7% |

**Table S2.** Pesticide residue reduction ratio (%) on the paper disc by the UV irradiation under the pAOP condition with 85 μmol/mol of ozone and UV_254_ at 60℃ for 24 h (tukey test, *p* < 0.05)

|  | Residue reduction ratio (%) | | | |
| --- | --- | --- | --- | --- |
|  | UV_254_ irradiation | | | |
|  | 0.9 W/m^2^ | 1.6 W/m^2^ | 3.3 W/m^2^ | 4.3 W/m^2^ |
| Carbendazim  Dimethomorph  Fluquinconazole  Imidacloprid  Myclobutanil  Tetraconazole  Thiamethoxam | 40±2.5^a^  95±1.8^a^  87±4.1^a^  95±3.5^a^  92±1.5^a^  90±3.7^a^  96±3.1^a^ | 44±2.6^ab^  97±0.1^a^  92±4.0^ab^  97±1.9^a^  94±2.2^a^  93±2.5^a^  96±1.4^a^ | 49±3.4^b^  98±1.2^a^  93±1.2b^b^  97±1.0^a^  98±0.9^b^  94±2.8^a^  97±2.3^a^ | 52±2.2^b^  98±0.8^a^  94±1.1^b^  97±2.4^a^  98±1.4^b^  95±2.6^a^  98±1.6^a^ |

**Table S3.** Regression coefficient (β), coefficient of determination (R^2^), and F-test value of the predicted second-order polynomial models for the pesticide residue reduction by pAOP treatment.

| Pesticide | Intercept, X_0_ | Liner | | Quadratic | | | Cross Product | R^2^ | F-value (model) | F-value  (lack of fit) |
| --- | --- | --- | --- | --- | --- | --- | --- | --- | --- | --- |
|  |  | X_1_ | X_2_ | | X_1_^2^ | X_2_^2^ | X_1_X_2_ |  |  |  |
| Carbendazim | 21.08^***^ | 10.00^***^ | 7.34^***^ | | 3.30^***^ | 0.77 | -2.83^***^ | 0.9732 | 152.56^***^ | 0.54 |
| Difenoconazole | 63.56^***^ | 9.17^***^ | 6.17^***^ | | 2.17 | 0.17 | -0.75 | 0.8085 | 17.74^***^ | 0.39 |
| Dimethomorph | 52.89^***^ | 14.83^***^ | 11.17^***^ | | -3.50 | -0.17 | -1.00 | 0.9119 | 43.46^***^ | 1.20 |
| Fludioxonil | 90.15^***^ | 2.39 | 2.39 | | -0.72 | 0.28 | 0.17 | 0.2549 | 1.44 | 0.07 |
| Fluquinconazole | 31.44^***^ | 4.944^***^ | 6.861^***^ | | 1.00 | -2.58 | 3.00^**^ | 0.9042 | 39.64^***^ | 0.25 |
| Imidacloprid | 70.70^***^ | 5.22^**^ | 3.33 | | 1.78 | 0.78 | 1.00 | 0.5404 | 4.94^**^ | 0.02 |
| Myclobutanil | 41.30^***^ | 5.94^***^ | 7.28^***^ | | -0.61 | 2.06 | 3.50 | 0.8014 | 16.95^***^ | 0.31 |
| Pyrimethanil | 34.07^***^ | 2.83^**^ | 5.22^***^ | | 0.39 | 0.89 | -0.25 | 0.6872 | 9.23^***^ | 0.17 |
| Tebuconazole | 42.59^***^ | 8.39^***^ | 5.67^***^ | | 5.94^**^ | 1.44 | 3.33^*^ | 0.8278 | 20.19^***^ | 0.58 |
| Tetraconazole | 51.04^***^ | 2.50 | 7.22^***^ | | 0.94 | 0.78 | 3.08 | 0.6334 | 7.26^***^ | 0.20 |
| Thiamethoxam | 54.89^***^ | 12.61^***^ | 9.00^***^ | | 6.83^**^ | 0.33 | -1.08 | 0.9235 | 50.70^***^ | 0.28 |

X_1_ = ozone concentration (μmol/mol), X_2_ = treatment time (h); Level of significance * p < 0.05, ** p < 0.01, *** p < 0.001.

**Table S4**. The physicochemical properties of the tested pesticides

| Pesticide | Boiling point  (℃) | Vapor pressure  (mPa, 25℃) | Henry’s law constant  (Pa m^3^ mol^-1^, 25℃) |
| --- | --- | --- | --- |
| Carbendazim | 305 (Decomp.^b^) | 1.5$\times{10}^{-1}$ | 3.6$\times{10}^{-3}$ |
| Difenoconazole | 101 | 3.33$\times{10}^{-5}$ | 9.0$\times{10}^{-7}$ |
| Dimethomorph | 280 (Decomp.) | *(E)* 9.7$\times{10}^{-4}$  *(Z)* 1.0$\times{10}^{-3}$ | *(E)* 5.4$\times{10}^{-6}$  *(Z)* 2.5$\times{10}^{-3}$ |
| Fludioxonil | 306 (Decomp.) | 3.9$\times{10}^{-4}$ | 5.4$\times{10}^{-5}$ |
| Fluquinconazole | 320 (Decomp.) | 6.4$\times{10}^{-6}$ | 2.09$\times{10}^{-6}$ (20℃) |
| Imidacloprid | 230 (Decomp.) | 4.0$\times{10}^{-7}$ (20℃) | 1.7 $\times{10}^{-10}$ |
| Myclobutanil | 391 | 1.98$\times{10}^{-1}$ (20℃) | 4.33$\times{10}^{-4}$ |
| Pyrimethanil | 190 (Decomp.) | 1.1 | 2.2$\times{10}^{-3}$ |
| Tebuconazole | 350 (Decomp.) | 1.3$\times{10}^{-3}$ | 1.0$\times{10}^{-5}$ |
| Tetraconazole | 235 (Decomp.) | 1.8$\times{10}^{-1}$ | 3.6$\times{10}^{-4}$ |
| Thiamethoxam | 147 (Decomp.) | 6.6$\times{10}^{-6}$ | 4.7$\times{10}^{-10}$ |

^a^ The data was obtained from The Pesticide Manual: A World Compendium (ed. 15) and International Union of Pure and Applied Chemistry (IUPAC) Pesticide Properties DataBase (https://sitem.herts.ac.uk/aeru/iupac/index.htm. Accessed on 18 Jan. 2021). ^b^ Decomposition temperature (℃)

**Table S5.** The tested pesticides for residue reduction in red pepper.

| Pesticide | Subgroup | MOA | A.I ^a)^  (%) | Formulation | Dilution folds |
| --- | --- | --- | --- | --- | --- |
| Carbendazim | Benzimidazole | Systemic | 60 | WP^b)^ | 2000 |
| Difenoconazole | Triazole | Systemic | 10 | WG^c)^ | 1000 |
| Dimethomorph | Morphorine | Systemic | 25 | WP | 1000 |
| Fludioxonil | Phenylpyrrole | Non-systemic | 20 | SC^d)^ | 2000 |
| Fluquinconazole | Triazole | Systemic | 6 | WP | 1000 |
| Imidacloprid | Neonicotinoid | Systemic | 8 | SC | 2000 |
| Myclobutanil | Triazole | Systemic | 10 | SC | 1500 |
| Pyrimethanil | Anilinopyrimidine | Systemic | 30 | WP | 1000 |
| Tebuconazole | Triazole | Systemic | 25 | WP | 2000 |
| Tetraconazole | Triazole | Systemic | 13 | EW^e)^ | 2000 |
| Thiamethoxam | Neonicotinoid | Systemic | 10 | WG | 2000 |

^a)^ Active ingredient; ^b)^ Wettable powder; ^c)^ Water dispersible granule; ^d)^ Suspension concentrate; ^e)^ Emulsion in water

**Table S5.** GC-MS instrumental condition for the analysis of fludioxonil, myclobutanil, tebuconazole, and tetraconazole.

| Instrument | Shimadzu QP-2010 GC-MS | | | | |
| --- | --- | --- | --- | --- | --- |
| Column | Rtx-5MS (0.25 μm, 0.25 mm x 30 m) | | | | |
| Carrier gas | Helium | | | | |
| Colum flow | 1.0 mL/min | | | | |
| Injection volume | 1 μL (Splitless) | | | | |
| Inlet temp. | 260 ℃ | | | | |
| Oven program | Rate (℃/min) | | Temp. (℃) | | Hold (min) |
|  | Initial | | 100 | | 2 |
|  | 4 | | 280 | | 7 |
| Ion source temp. | 230 ℃ | | | | |
| Interface temp. | 280 ℃ | | | | |
| Compound | Quantitative ion | Qualitative ion | | Retention time (min) | |
| Fludioxonil | 179 *m/z* | 150, 152 *m/z* | | 33.1 | |
| Myclobutanil | 248 *m/z* | 182, 249 *m/z* | | 33.4 | |
| Tebuconazole | 250 *m/z* | 163, 252 *m/z* | | 37.2 | |
| Tetraconazole | 336 *m/z* | 337, 338 *m/z* | | 29.1 | |

**Table S6.** LC-MS/MS Instrumental conditions for the quantitative analysis of the pesticides

| Instrument | Agilent Technologies 6460 Triple Quad LC/MS (USA) | | | | | |  |
| --- | --- | --- | --- | --- | --- | --- | --- |
| Column | Poroshell 120 EC-C18 (2.1 X 100mm, 2.7um), Agilent (USA) | | | | | |  |
| Column temp. | 35 ℃ | | | | | |  |
| Flow | 0.3 mL/min | | | | | |  |
| Injection volume | 1 μL | | | | | |  |
| Ion source | ESI (+) | | | | | |  |
| Mobile phase | A : 0.1% Formic acid, 5mM ammonium formate (in water),  B : 0.1% Formic acid, 5mM ammonium formate (in methanol) | | | | | |  |
|  | Time (min) | | A (%) | | B (%) | |  |
|  | 1.0 | | 85 | | 15 | |  |
|  | 1.5 | | 40 | | 60 | |  |
|  | 10.0 | | 10 | | 90 | |  |
|  | 12.0 | | 10 | | 90 | |  |
|  | 12.1 | | 2 | | 98 | |  |
|  | 16.0 | | 2 | | 98 | |  |
| **Mass spectrometer conditions** | | | | | | | |
| Ion source | | ESI (+) | | | | | |
| Compound | | MRM transition (*m/z*) | | Collision energy (CE) | |  |  |
| Carbendazim | | 192 > 160 (quantitative)  192 > 132 (qualitative) | | 25 V  40 V | |  |  |
| Difenoconazole | | 406 > 251 (quantitative)  406 > 337 (qualitative) | | 34 V  25 V | |  |  |
| Dimethomorph (E/Z) | | 388 > 301 (quantitative)  388 > 165 (qualitative) | | 20 V  25 V | |  |  |
| Fluquinconazole | | 376 > 307 (quantitative)  376 > 108 (qualitative) | | 30 V  69 V | |  |  |
| Imidacloprid | | 256 > 209 (quantitative)  256 > 175 (qualitative) | | 20 V  20 V | |  |  |
| Pyrimethanil | | 200 > 107 (quantitative)  200 > 82 (qualitative) | | 20 V  25 V | |  |  |
| Thiamethoxam | | 292 > 211 (quantitative)  292 > 181 (qualitative) | | 10 V  20 V | |  |  |

**Table S7.** Instrumental condition for analysis of capsaicin and dihydrocapsaicin in the pepper.

| Instrument | Thermo Scientific UltiMate 3000 HPLC | | |
| --- | --- | --- | --- |
| Column | Agilent XDB-C18 (5 μm, 4.6 x 250 mm) | | |
| Column temp. | 30 ℃ | | |
| Flow | 1.0 mL/min | | |
| Injection vol. | 10 μL | | |
| Wavelength | 280 nm | | |
| Mobile phase | A : Acetonitrile, B : Water | | |
| Gradient program | Time (min) | A (%) | B (%) |
|  | 0 | 45 | 55 |
|  | 20 | 50 | 50 |
|  | 25 | 95 | 5 |
|  | 31 | 95 | 5 |


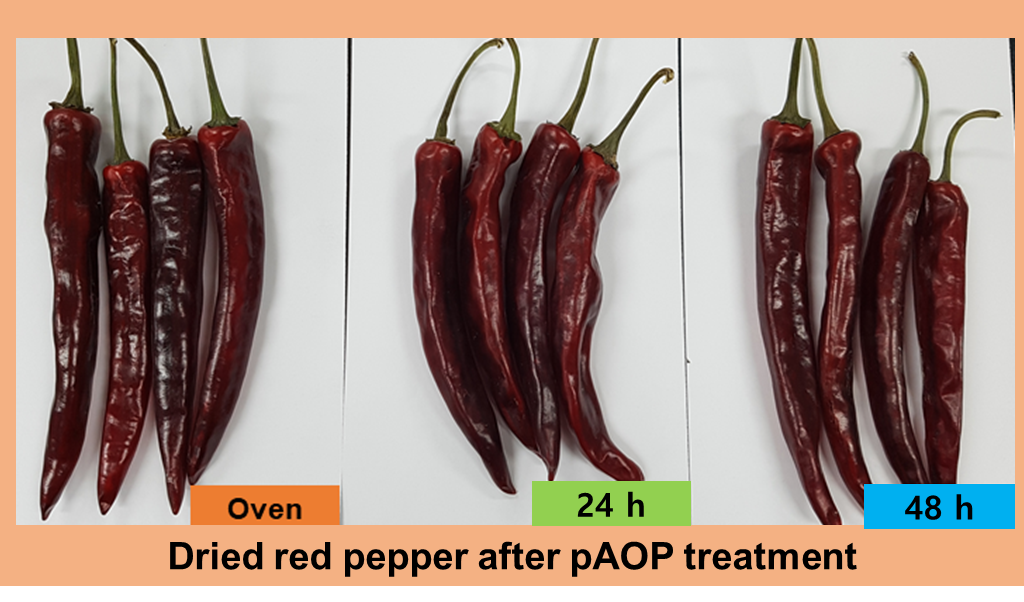


**Figure S1**. Dried pepper after the pAOP treatment with 24 μmol/mol ozone and 9.6 W m^-2^ for 48h
